# Use of Cement Suspension as an Alternative Matrix Material for Textile-Reinforced Concrete

**DOI:** 10.3390/ma14092127

**Published:** 2021-04-22

**Authors:** Richard Fürst, Eliška Fürst, Tomáš Vlach, Jakub Řepka, Marek Pokorný, Vladimír Mózer

**Affiliations:** 1 Fire Laboratory, University Centre for Energy Efficient Buildings of Czech Technical University in Prague, Třinecká 1024, 273 43 Buštěhrad, Czech Republic; marek.pokorny@cvut.cz (M.P.); vladimir.mozer@cvut.cz (V.M.); 2 Faculty of Civil Engineering, Czech Technical University in Prague, 166 29 Prague 6, Czech Republic; tomas.vlach@cvut.cz (T.V.); jakub.repka@cvut.cz (J.Ř.); 3 Federal Institute for Materials Research and Testing (BAM), Division 7.3-Fire Engineering, Unter den Eichen 87, 12205 Berlin, Germany; 4 Institute of Organic Chemistry and Biochemistry of the Czech Academy of Sciences, Flemingovo n. 2, 16610 Prague 6, Czech Republic; eliska.fuerst@mdc-berlin.de; 5 Allosteric Proteomics, Max Delbrück Center for Molecular Medicine, Robert-Rössle Straße 10, 13125 Berlin, Germany; 6 Laboratory of Composite Structures, University Centre for Energy Efficient Buildings of Czech Technical University in Prague, Třinecká 1024, 273 43 Buštěhrad, Czech Republic

**Keywords:** textile-reinforced concrete, non-combustibility, cohesion, high-performance concrete, carbon fibers, cement matrix

## Abstract

Textile-reinforced concrete (TRC) is a material consisting of high-performance concrete (HPC) and tensile reinforcement comprised of carbon roving with epoxy resin matrix. However, the problem of low epoxy resin resistance at higher temperatures persists. In this work, an alternative to the epoxy resin matrix, a non-combustible cement suspension (cement milk) which has proven stability at elevated temperatures, was evaluated. In the first part of the work, microscopic research was carried out to determine the distribution of particle sizes in the cement suspension. Subsequently, five series of plate samples differing in the type of cement and the method of textile reinforcement saturation were designed and prepared. Mechanical experiments (four-point bending tests) were carried out to verify the properties of each sample type. It was found that the highest efficiency of carbon roving saturation was achieved by using finer ground cement (CEM 52.5) and the pressure saturation method. Moreover, this solution also exhibited the best results in the four-point bending test. Finally, the use of CEM 52.5 in the cement matrix appears to be a feasible variant for TRC constructions that could overcome problems with its low temperature resistance.

## 1. Introduction

Textile-reinforced concrete (TRC) is a construction material currently used for non-load bearing structures [1,2,3,4,5] and facade panels, but it could potentially be used in load-bearing construction elements [6,7]. In load-bearing structures, marginal TRC application issues arise from the ability to withstand high temperatures during fire. In previous work the main weakness of TRC was identified as the behavior of the homogenized carbon-fiber reinforcement with epoxy resin matrix at elevated temperatures [8,9]. Fire experiments showed that the load-bearing capacity of TRC at elevated temperatures depends on the interaction between the textile reinforcement and high-performance concrete (HPC). Since epoxy resins used in this variant of TRC have low resistance to higher temperatures [10,11,12,13], this study focused on the identification of an alternative available material for the homogenization of textile reinforcement before installation in concrete.

**Figure 1:**
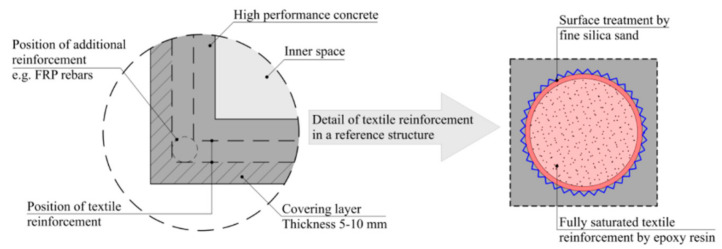
Composition of a TRC structural column.

The traditional variant of carbon TRC is composed of impregnated carbon roving HPC with a synthetic resin matrix [14]. By replacing the steel reinforcing elements (rebars, wires etc.) of standard reinforced concrete structures with impregnated carbon roving, corrosion protection is not required and the covering layer can be significantly reduced. The combination of HPC with carbon-fiber reinforcement allows for subtler structural elements. A cross-section indicating general composition of a TRC structure, a hollow column, is shown in Figure 1.

Carbon-fiber roving impregnation brings higher load-bearing capacity due to the increased tensile load distribution across the entire cross-section of the carbon fiber strand. The epoxy resin which is used as impregnation material, however, increases the brittleness of the composite. Synthetic-based materials are increasingly being used in modern construction, often in the form of synthetic epoxy resins and adhesives. However, in load-bearing structures, this material can negatively affect their mechanical properties from the fire safety perspective. For these structures, it is necessary to determine their behavior at elevated temperatures, and identify appropriate protective measures based on these findings. In composite structures, their load-bearing capacity depends on the interaction between the synthetic materials and textile reinforcement.

**Figure 2:**
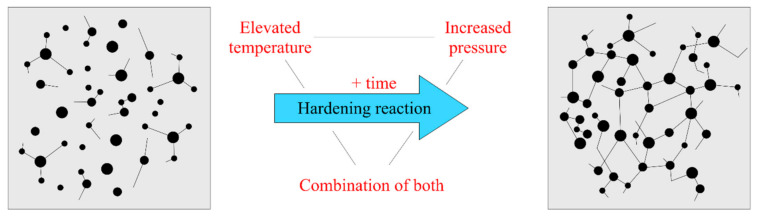
Diagram of a resin hardening process.

In general, the synthetic resins consist of a two-component resin and a hardener. According to the specified preparation instructions, resins change their structure from a free molecular arrangement to a stable, crosslinked mesh-state. After crosslinking, the epoxy resin acquires its final mechanical properties. This process is called curing (Figure 2).

The standard hardening process includes elevated pressure and temperature, or a combination thereof, for a prescribed time. The specific method depends on the selected product. For some types of resins e.g., [15], better mechanical properties or higher temperature resistance are achieved through a slower and longer hardening process. These properties also depend on the molecular weight, the distribution of the macromolecules in the resin, and the phase state, among others [10]. It is necessary to consider the method of the hardening process. For example, in the production of TRC, it is not possible to use resins with higher temperature resistance, because the process of hardening would be overly complicated and financially demanding [16].

**Figure 3:**
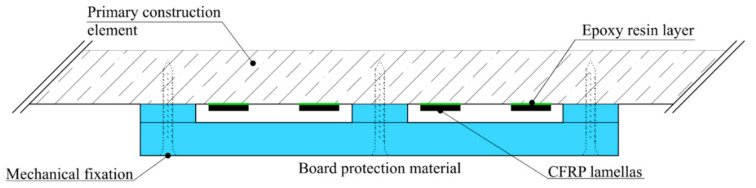
Protection of CFRP (Carbon-fiber-reinforced polymer) lamellas against elevated temperature in epoxy resin layers by board material.

Another example of epoxy resin use is found in carbon lamellas, in the form of the additional external tensile reinforcement of existing structures. Without additional fire protection, the lamellas would achieve practically zero fire resistance, and the affected structure would be significantly weakened. In this case, it is relatively easy to design additional protection against elevated temperature, e.g., from board materials (Figure 3) [17].

An additional possible application of epoxy resins in concrete structures could be in the reparation of damaged concrete structures [18], the modification of concrete surfaces, and the elimination of cracks on concrete surfaces [19]. The other possibility of using synthetic resins is in the improvement of mortar-based building materials [20].

A problem occurs when TRC is considered as a material for potential use in load-bearing constructions. From the perspective of fire protection, textile reinforcement is a combination of materials in which the loss of interaction between the materials is probable. Synthetic resins play a crucial role in TRC by redistributing tension to the entire strand of textile reinforcement. When the temperature rises to the synthetic resin glazing temperature, a massive increase in deformation, decrease in modulus of elasticity, and loss of interaction occurs. Several studies at elevated temperatures have dealt with the subject of textile-reinforced concrete [21]. In general, it can be considered that synthetic resins are advantageous for the tensile reinforcement matrix because their homogeneous structure allows for the full saturation of the textile reinforcement. At the same time, resins provide additional protection against mechanical damage during production and from atmospheric corrosion [22]. However, the wider use of these structures can be affected by their instability at elevated temperatures. The use of synthetic resins in the production of TRC is currently satisfactory at room temperatures, where an excellent interaction between the materials is achieved. The protection of TRC against loss of interaction is possible either by additional fire protection, as in the case of carbon lamellas, or by the design of a matrix material with sufficient temperature resistance higher than 700 °C. However, the advantage of not using additional fire protection is maintaining the subtle character of the TRC structure and its high visual quality.

Considering all these problematic areas of TRC with an epoxy resin matrix at elevated temperatures, an alternative material solution was sought to potentially substitute flammable epoxy resin, as it has been shown that this component contributes to the development of fire [8,23].

In reaction to the temperature instability of an organic matrix, several studies are examining how to replace the flammable organic matrix of textile reinforcement. One possible variant is to use textile fibers as a reinforcement layer in the mortar, which improves the mechanical properties of masonry [24]. Other studies investigated the influence of the inorganic matrix materials on the mechanical properties if textile reinforcement while using materials such as SiO_2_, nano-silica, or micro-silica [25,26,27,28]. Simultaneously, cement suspensions and cement mixtures are also used. In the case of cement mixture for the impregnation of textile reinforcement, the combination of cement, micro silica, plasticizer, and water has been discussed [29]. In a study describing mechanical properties with impregnation of glass textile reinforcement, cement suspension with cement CEM 42,5 was used [30]. All these studies describe improvements to the mechanical properties of textile reinforcements, and explain the influence of particle size on the depth of impregnation, but these studies dealt mainly with glass reinforcement. However, it is necessary to consider which types of textile reinforcement are more beneficial. Therefore, it is advisable to investigate the matrix alternative with carbon fiber because, in contrast with the individual glass fiber filaments, the carbons filaments are half the size [31].

Based on the mechanical properties and mechanical parameters (especially the size of the individual carbon filaments), it is appropriate to determine how these materials could be used with cement suspension impregnation. For this reason, a series of experiments have been proposed to verify the behavior of commonly available types of cement, as an obtainable and common material, in impregnating carbon reinforcement with the most straightforward possible application.

**Figure 4:**
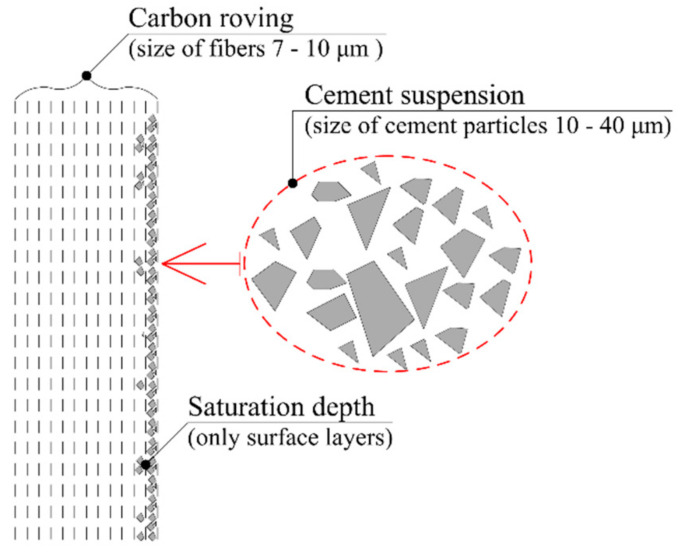
Scheme of carbon roving with cement suspension.

This solution would eliminate the low resistance of TRC structures to elevated temperatures [29]. On the other hand, a problem in the curing process could emerge. The size of the cement particles in the cement suspension is larger than the size of filaments in the carbon roving. The distribution of the cement particle size in cement generally ranges from 8 to 40 µm [32,33]. These particles might not penetrate the deeper layers of textile reinforcement, and the required interaction between the textile reinforcement and HPC may not occur (Figure 4).

The TRC presented in this paper was developed at the Faculty of Civil Engineering, Czech Technical University (CTU), in Prague, and at the University Centre for Energy Efficient Buildings (UCEEB), CTU, in Prague. Following the development of this material, an alternative matrix of textile reinforcement was projected to verify this solution and evaluate the possibility of future use of cement suspension as an alternative material for textile reinforcement.

This work focuses on an alternative textile reinforcement matrix material because, from the viewpoint of fire protection, an epoxy resin matrix could theoretically lead to the collapse of an entire TRC construction [12,34,35]. Therefore, a cement suspension matrix was proposed which eliminates all flammable components of this composite material. Various methods of cement suspension application and depth of textile reinforcement saturation were investigated, based on the cement type. Mechanical properties were determined by four-point bending test and compared with traditional TRC samples with an epoxy-resin matrix, which served as a positive control of the full-roving saturation. These tests were conducted at standard temperatures. Different cement types were also investigated from the viewpoint of cement particle distribution.

## 2. Materials and Methods

### 2.1. High-Performance Concrete Mixture

The HPC mixture used for the preparation of experimental samples was prepared according to Table 1. This HPC mixture was developed at the Czech Technical University in Prague, Czech Republic. By following the prescribed procedure, this material can reach a compressive strength of about 140.5 MPa according to [36], a tensile strength of 15.4 MPa according to [37], and a modulus of elasticity value of about 49.5 GPa [4].

### 2.2. Textile Tensile Reinforcement

The carbon roving used for tensile reinforcement was developed by the Tenax company (Viganò (LC), Italy) as a commercially available product named Tenax STS40 F13 24K 1600tex. The material properties of the carbon roving are described in Table 2. Two types of cement were used for the subsequent saturation of the textile reinforcement: specifically, CEM 42.5 R and CEM 52.5 R.

### 2.3. Geometry of Experimental Samples

**Figure 5:**
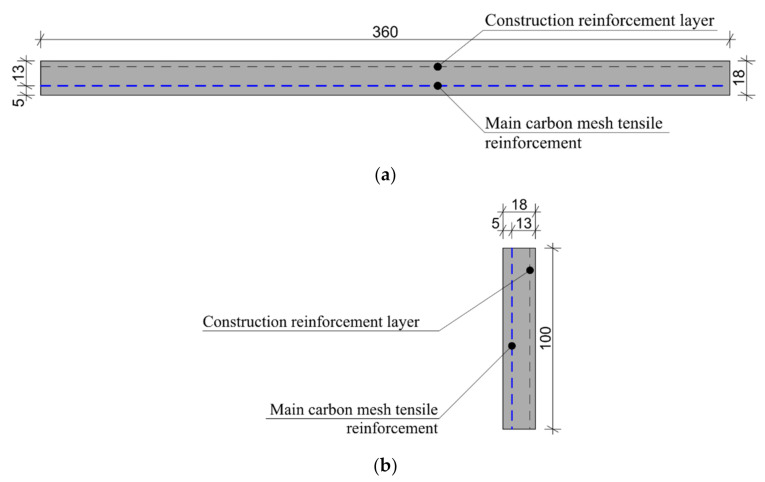
Description of the experimental sample and position of reinforcement mesh (unit: mm): (**a**) Plan view of the experimental sample; (**b**) Cross-section of the experimental sample.

For the mechanical experiment, the experimental sample parameters were chosen according to [37,38], with modified specimen height. The distance between the centers of support was standard for a four-point bending test: 300 mm and 100 mm between loading support centers. The experimental plate samples were designed with dimensions of 100 mm × 360 mm × 18 mm and provided with two layers of textile reinforcement. The upper layer of reinforcement mesh was used only for construction reasons. Thus, only the reinforcement on the bottom bend side was considered as the primary tensile reinforcement. In a real construction, specimens are logically able to transmit a bend loading in both directions. The dimensions of the experimental sample and positions of its reinforcement mesh are depicted in Figure 5. These experimental sample shapes and sizes was also chosen based on their suitability for the subsequent verification of mechanical properties in four-point bending tests. The thickness of the sample was adjusted to the approximate thickness of the TRC in real applications, and was also based on previous experiments [39,40]. Then, it was possible to compare the results. The bending moment in between the loading supports was constant. This was the monitored area where the cracks development and the collapse of the sample occurred. The mechanical-resistance experiments were conducted at standard (room) temperature.

**Figure 6:**
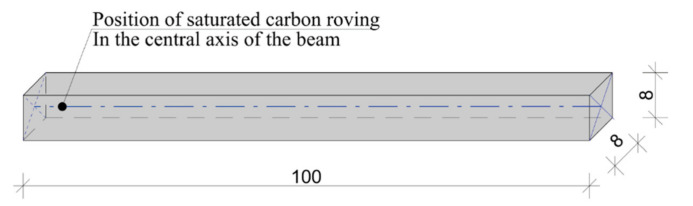
Description of the beam sample with the position of the saturated roving (unit: mm).

In the first part of sample manufacturing, the rovings were saturated using the methods described in Table 3. After curing, they were fixed in silicon form and placed into a concrete beam with a length of 100 mm and cross-section area of 8 mm × 8 mm. This process minimalized the risk of damaging the saturated fibers during cutting. These beam samples could be then cut without fiber breakage or defibration, and subjected to microscopy analysis. The geometry of experimental sample is displayed in Figure 6.

### 2.4. Microscopy

CEM 42.5 R and CEM 52.5 R samples were weighed on analytical scales and supplemented with water so that both suspensions contained the same quantity of particles in the specified volume. From both samples, 50 μL of suspension were taken and analyzed on a Zeiss LSM 780 (Carl Zeiss AG, Oberkochen, Germany) confocal microscope using the basic optical microscope settings and 100× magnification. The microscope photographs were analyzed using Cell Profiler 4.0.4. All analyzed samples were taken from three independent replicates for each type of cement suspension.

Images of beam samples were taken with a Canon EOS 5D Mark II camera (Canon Inc, Óta, Japan) and the saturation depth was further analyzed using ImageJ, version 1.8.0.

### 2.5. Preparations of Experimental Samples

The development of the experimental samples comprised of textile reinforcement saturation in tension frames, preparation of production forms with the required size, and conditioning. Altogether, six experimental sample variants were developed in triplicate. Each sample was labelled with a serial letter (A-E) and a sample number (1-3) (Table 3).

The experimental samples of series A and B were saturated under pressure to achieve a deeper saturation of the carbon filaments. The process of saturation under increased pressure was further enhanced by the application of a significantly excessive amount of cement suspension, which was mechanically pressed into the roving. During this process, the impregnated fibers were at the same time tensioned on a tension frame and left to harden to form a solid reinforcing mesh.

**Figure 7:**
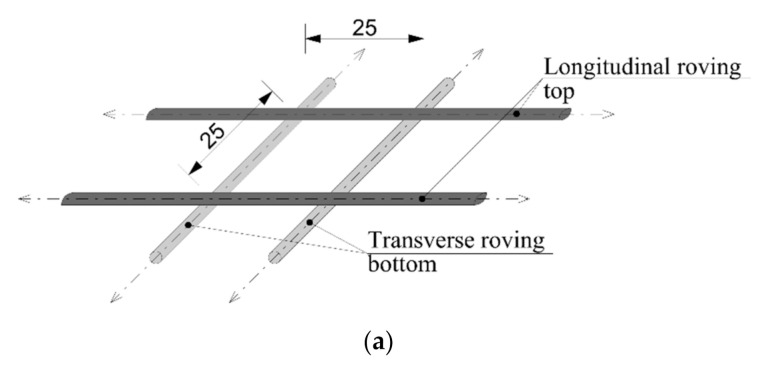

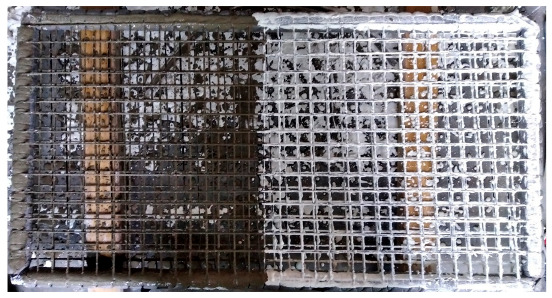
Preparation of reinforcement mesh: (**a**) Method of carbon mesh preparation (unit: mm); (**b**) Mesh from carbon roving saturated with cement suspension–surface application by roller (from left): CEM 42.5 R, CEM 52.5 R.

In the samples of series C and D, the carbon fibers were tensioned on a tension frame. The layer of cement suspension was subsequently applied by using a roller. The fibers were tensioned on a tension frame in two layers. In the first layer, all the rovings were positioned in a longitudinal direction; and in a transverse direction in the second layer. In this case, the joints of the fibers occurred only at the fiber overlaps. However, according to [41], these joints do not affect the resulting tensile strength. The final form of the reinforcement mesh was completed with a 25 mm distance between the fibers. The final reinforcement mesh with hardened cement layers and the method of forming are described in Figure 7.

**Figure 8:**
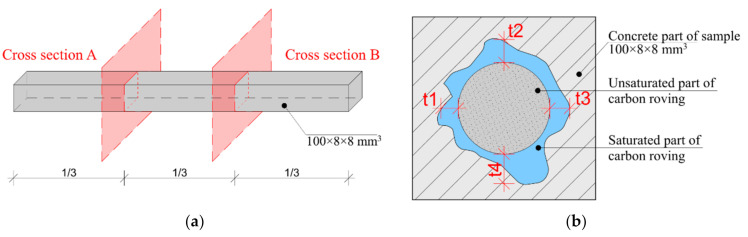
Experimental beam samples: (**a**) Scheme of experimental samples with cross-section position; (**b**) Method of the saturation depth evaluation.

Each experimental sample was conditioned for 28 days in a lime–water bath [37]. The beam samples were cut at one third of length and visually analyzed as described in Section 2.4, for the evaluation of the overall depth and percentage of saturation (Figure 8).

### 2.6. Statistical Data Analysis

For the statistical evaluation of the saturation depth data (Figure 12), the so-called expanded uncertainty coefficient *k*_u_ = 2.0 (interval 95% of the contained values) was used. In other statistical assessments, outliers were not considered. Values outside the 90% quantile range (10% of outlying values from top and bottom) were considered as outliers. For the statistical evaluation of cement particles distribution, a Student’s t-test was used. All statistical analyses were performed in GraphPad Prism, version 8.0, and MS Excel, 2019. The four-point bending tests were performed in a Galbadini Quasar at 10 kN. According to device technical sheet, the measurement accuracy was ±1% (accuracy class 1) from the maximal load-cell value, in accordance with EN ISO 7500/1.

## 3. Results

### 3.1. Particle Size Distribution in Cement Suspension

**Figure 9:**
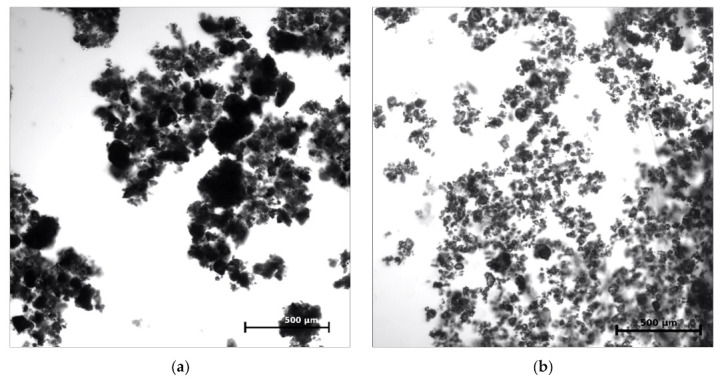
Microscope pictures of cement suspension: (**a**) CEM 42.5 R; (**b**) CEM 52.5 R.

**Figure 10:**
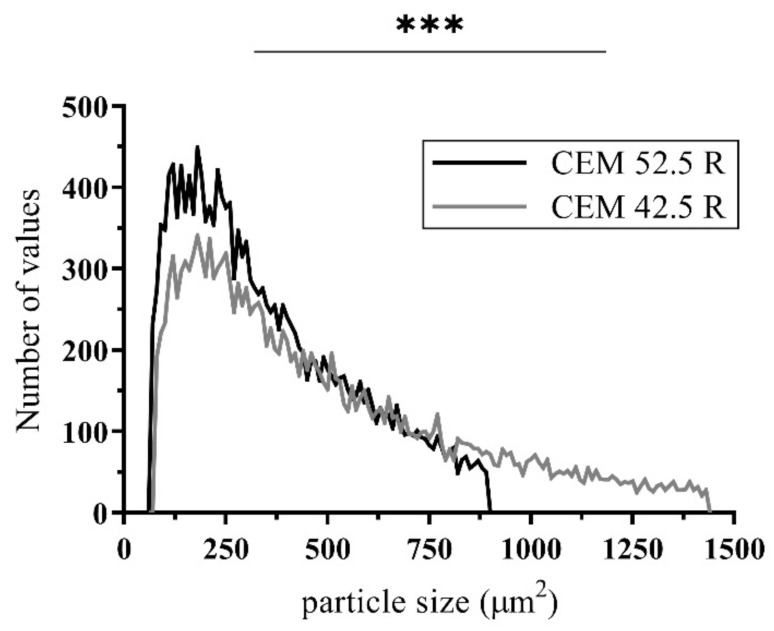
Comparison of size and distribution of cement particles in cement suspensions. A Student’s *t* test was used for statistical testing; *** *p* < 0.001.

In the first part of the assessment, cement particles were imaged using confocal microscopy. The distribution and size of the CEM 42.5 R and CEM 52.5 R particles were compared. Overall, 30 microscope photographs from three independent samples for each cement type were analyzed and mutually adjusted based on the numbers of analyzed particles, so that both examined samples contained the same number of particles (in total more than 20,000). From the microscope photographs (Figure 9), it is clear that CEM 52.5 R had overall better distribution in cement suspension, while CEM 42.5 R contained more particles bound together in the so-called floccules, which may cause a problem of insufficient carbon filament saturation (3.2). Moreover, the size distribution of cement particles differed for the two types of cement. CEM 52.5 R had a median of a particle size 301.5 μm^2^ (corresponding to the mean value of one-side length of 9.8 μm), while the median of the particle size in CEM 42.5 R was 397.8 μm^2^ (which was 11.3 μm in length). These measurements also correspond to the findings mentioned in [42]. When also considering the formation of floccules in CEM 42.5 R, the results indicate an overall poorer probability of deep carbon fibers saturation. From the frequency distribution chart in Figure 10, it follows that not only the median size of particles, but also the number of larger particles overall was less in CEM 52.5 R than in CEM 42.5 R (*p* < 0.0001).

### 3.2. Saturation Depth of Beam Samples

Next, the hypothesis that the penetration of carbon roving strongly depends on cement type and saturation method was tested. An identical series of samples (A–E) was produced as described in Table 3, but in the shape of beam samples (Figure 6). The beams were cut in three parts. On each cross-section cut, the depth of saturation was measured (Figure 8).

The reason for using two different types of cement was to determine the effect of particle size on roving saturation depth. In the case of the epoxy resin matrix, the fibers were fully saturated by penetration from the surface (application by a roller). This was caused by the homogeneous structure of the resin. Two cement types with an overall higher quantity of small particles were chosen: CEM 52.5 R and CEM 42.5 R. As anticipated, the smaller cement particles could penetrate deeper and achieve a better interaction between the HPC and the tensile reinforcement. The white variant of CEM 52.5 R was chosen because of the easier subsequent identification of the saturation depth.

**Figure 11:**
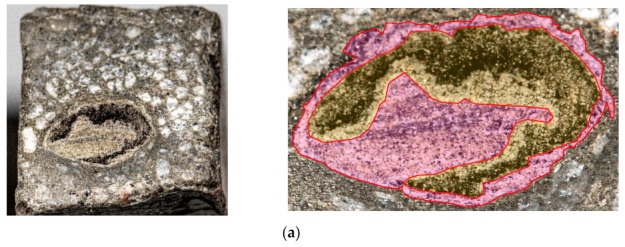

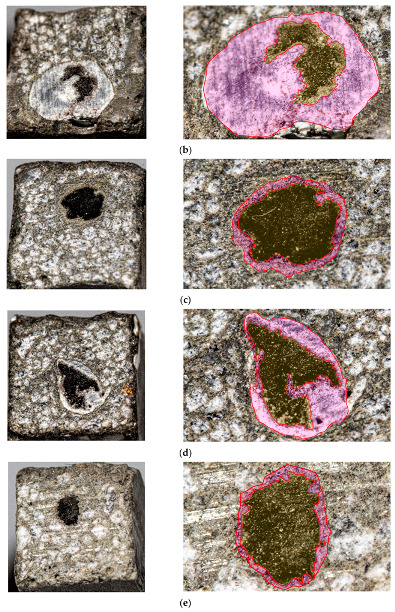
Cross-sections of beam samples. Left panels: whole cross-section area of beam sample. Right panels: detailed view of the cross-section area with the saturated part of the carbon roving in red and the non-saturated carbon roving in yellow: (**a**) Experimental series A—pressure saturation CEM 42.5 R; (**b**) Experimental series B—pressure saturation CEM 52.5 R; (**c**) Experimental series C—surface saturation by a roller CEM 42.5 R; (**d**) Experimental series D—surface saturation by a roller CEM 52.5 R; (**e**) Experimental series E—without saturation.

It is apparent from the photographs, when comparing the different methods of saturation and different types of cement, that the best results were obtained in the series A and B samples (pressure saturation method). Greater saturation depth was reached in series B samples, supporting the hypothesis that smaller particles penetrate the roving better. Detailed images of all beam samples are provided in Figure 11.

Unfortunately, after the hardening of the concrete mixture in sample E (natural penetration of cement suspension from the wet concrete mixture), it turned out that the number of cement particles penetrating the beam core was not sufficient (Figure 11). Most of the concrete mixture particles were only on the surface of the carbon roving, and the saturation was inadequate. The reason for the poor saturation of the carbon fibers with concrete mixture was the size of the particles in the mixture. Generally, the particle size median was approximately 9 μm (CEM 52.5 R) and 12 μm (CEM 42.5 R) (Figure 10). Due to the size of each carbon roving filament (7–10 μm), the cement mixture particles could not reach the core of the carbon roving. Therefore, there was not enough of the cement material in the core of the carbon roving, and the filaments were not fully activated.

**Figure 12:**
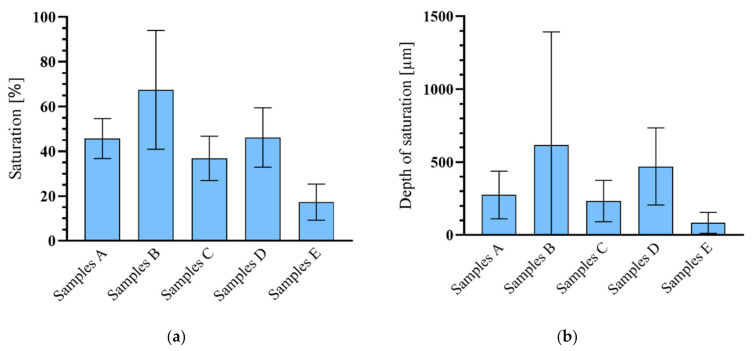
Evaluation of microscopic research of textile reinforcement saturation: (**a**) Fiber saturation expressed as a percentage; (**b**) Average depth of textile reinforcement saturation. Legend: Samples A—Experimental samples saturated with CEM 42.5 R using pressure saturation; Samples B—Experimental samples saturated with CEM 52.5 R using pressure saturation; Samples C—Experimental samples saturated with CEM 42.5 R using surface saturation by a roller; Samples D—Experimental samples saturated with CEM 52.5 R using surface saturation by a roller; Samples E—Experimental samples without saturation.

A detailed chart of the average percent and saturation depth values is provided in Figure 12. It is obvious that the most effective method of saturation of textile reinforcement was pressure impregnation with cement suspension in CEM 52.5 R. A microscopy investigation determined that both the size of cement particles and the method of pressure saturation increased the saturation by 22% over that of cement CEM 42.5 R. In contrast, the increase in saturation between experimental samples C and D was only 9%. The type of cement used has an influence on the saturation depth (μm) in both methods of saturation. CEM 52.5 R was about 2.3 times better than CEM 42.5 R regarding saturation depth.

### 3.3. Four-Point Bending Tests

The mechanical test evaluation focused on verifying the influence of the cement suspension on performance by means of bending tests under four-point bending at normal temperature, according to [37]. As a reference, plate samples with an epoxy resin textile reinforcement matrix were used. The mechanical tests were terminated when the experimental sample was broken or when the trend of increasing values measured was stabilized. The four-point bending test was performed with a controlled constant load speed of 2.0 mm per minute.

**Figure 13:**
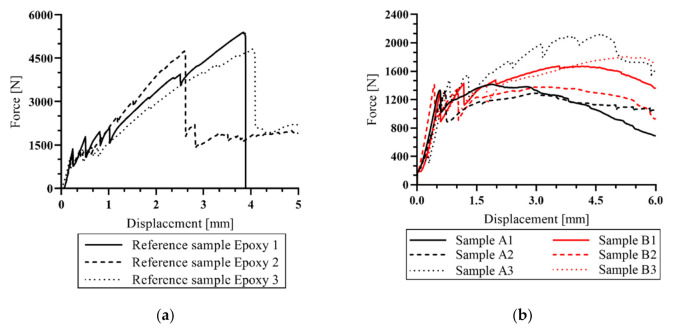
Measured data from four-point bending test: (**a**) Reference samples; (**b**) Comparison of sample series A and B.

From the following charts (Figure 13) it is evident that the first crack occurred in the interval from 1.1 to 1.6 kN. This value represents the tensile strength of the concrete part of the experimental sample. The course of behavior after the development of the first crack is essential, because it was found that the experimental sample (Figure 13b) is capable of further transmitting the tensile force. In the reference samples, there was evidence that after the first crack arose the force was still being transmitted to the tensile reinforcement before its tensile strength was exceeded. Subsequent progressive breakage of the sample was observed after exceeding approximately 4.7 kN.

In contrast, the other tested samples did not exceed the tensile strength and did not break. Samples of the A and B series showed a demonstrable reinforcement–concrete interaction, and the specimen retained the ability to at least partially transmit the bending force. The maximum achieved force of the A and B samples was only 28% of the capacity of the reference samples. The decreasing course of the force measured in samples of the A and B series began in the interval between 4–5 mm displacement, with the average force reaching 1.62 kN for series A and 1.63 kN for series B. However, only sample A3 from series A reached considerably higher values than the other samples from this series. After excluding sample A3, an average value of 1.35 kN was obtained. Nevertheless, series B was considered to be significant, as there was no considerable variance of the measured values, as was the case in series A.

**Figure 14:**
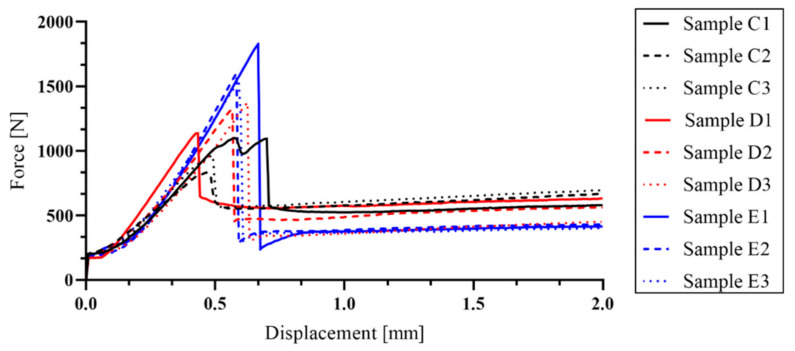
Course of values measured on samples from the C, D (surface saturation by a roller), and E (without any saturation) series.

During the mechanical tests, only one crack developed under the load support (samples C–E). In the reference samples, it was possible to observe a break of the sample, but in the rest of experimental samples (A–E series) the break did not occur because the tensile strength of the textile reinforcement was not exceeded. The formation of further cracks in the experimental samples was not observed, presumably because of the size and speed of crack development. Due to the increased deformation, the fibers of the textile reinforcement began to be pulled out from the sample (Figure 14).

## 4. Discussion

In a previous study, the main problematic properties of textile-reinforced concrete were described [8]. Although epoxy resin is an excellent material in the function of textile reinforcement matrix, its low resistance to elevated temperatures is problematic from the perspective of fire safety. Therefore, an alternative non-combustible matrix material that would naturally overcome the problem of low resistance at elevated temperatures was examined. A cement suspension matrix was used to verify its effect on the mechanical properties of the experimental samples at normal room temperature (20 °C). The mechanical properties measured in these samples were compared to values obtained from previous experiments and reference samples (Figure 13). Experimental samples in this work were made from high-performance concrete (HPC) with tensile reinforcement from carbon fibers.

In this study, the influence of the type of cement in the cement suspension as well as the type of textile reinforcement saturation procedure on the mechanical properties was examined. The saturation was performed in two different ways. The first method was mechanical pressure saturation (series A and B). The second was surface saturation using a roller (series C and D). The same procedure was performed with the epoxy resin matrix (reference series Epoxy 1, 2 and 3). Series E was developed as the negative control without any matrix, where additional saturation of the concrete mixture was not applied. Some minor penetration of the cement suspension from the wet concrete mixture was, however, present. Based on the literature and material review, it was suggested that the problem of insufficient saturation could have occurred because of the size of cement particles [43]. The standard type of cement consists mainly of particles whose size is generally larger than each carbon filament of the textile roving (>7 μm). Therefore, a microscopy examination describing the distribution of the size of the cement particles in the suspension was carried out. This showed that the CEM 52.5 R had the dominant share of cement particles smaller than those of CEM 42.5 R (Figure 10). Also, the number of larger particles (whose area was larger than 1000 µm^2^) was greater in CEM 42.5 R. All these findings led to the conclusion that CEM 52.5 R should have had better performance in the subsequent mechanical experiments, because of the higher probability of achieving a deeper saturation of the textile reinforcement. However, it is necessary to consider the real quantity of saturated filaments in the carbon roving. There is a possibility that, although the depth of saturation may be sufficient, the risk (mainly in CEM 52.5 R) of carbon filament swelling endures. The increase of filament diameter can be interpreted wrongly as deep saturation when only a small quantity of filaments have actually been activated for interaction. The dependence of the mechanical performance on the extent of saturation was confirmed in the mechanical tests, where the B samples demonstrated the best load capacity.

Four types of reinforcement mesh were prepared by using a different cement type in the cement suspension and a different method of saturation. Two types of experimental samples were developed, plate (100 mm × 360 mm × 18 mm) and beam (8 mm × 8 mm × 100 mm). The plate samples were used for the four-point bending tests while the beam samples were used for the evaluation of the saturation depth. As expected, a deeper saturation of the textile reinforcement was achieved using CEM 52.5 R. For experimental samples of the B series, the average saturation reached 67%. In contrast, series A reached an average saturation of only 45%. The other experimental samples exhibited significantly lower depth of saturation, e.g., in series C it was 36%, series D 46%, and in series E (lowest percent saturation caused by migration from the concrete mixture), only 17%. It should be pointed out that the method of application of the cement suspension was found to have a significant impact on saturation. The difference in saturation between series A and B was 21%; between C and D, it was 9%. This means that the influence of cement particle size is important in a case when the small particles are mechanically pressurized into the textile reinforcement. Using this method, the best overall results were achieved. With surface application using a roller, there was no significant difference in the depth of saturation between CEM 42.5 R and CEM 52.5 R.

After this evaluation, experimental samples were prepared for the comparison of the influence of saturation on bending capacity. The assessment of each experimental sample was divided into two parts. The first part of the assessment was before the first crack formation (where the tensile strength of the concrete was exceeded) and the second one was after the crack had occurred. However, the second part was essential for the evaluation, because it was possible to observe whether there was an interaction between the materials. In the E series samples (without additional cement impregnation) there was the lowest saturation of textile reinforcement (due to the larger particle size in the concrete mixture) and after the development of the first crack, there was no interaction between the materials. Insufficient interaction led to the lack of tensile load transmission to the carbon-fiber reinforcement. By contrast, for the C and D series, the cement suspension formed a layer on the surface of the textile fibers after penetration. After that, this hardened layer prevented the penetration of the concrete mixture into the textile reinforcement. Therefore, in series C and D, it was not possible to achieve good adhesion between the textile reinforcement and concrete, as only the surface application method was used. During the mechanical test, the thickness of the hardened suspension layer could not transfer the tensile stress to the tensile reinforcement.

Therefore, even in samples C and D, the interaction between the materials was not sufficient. Only in the series A and B samples was there sufficient saturation of the textile reinforcement. It was found that in a pressure saturation, the concrete mixture did not penetrate all the layers of textile reinforcement to a full extent. Nevertheless, the depth of impregnation was enough to cause sufficient interaction (load transfer) between the materials. As shown in Figure 11, it was possible to observe an increasing trend of the load curve in the A and B series after the formation of the first crack. This confirmed the similar trend of the particle size in the impregnation mixture, which was described on glass fibers in [24,25,26,27,28,29].

**Figure 15:**
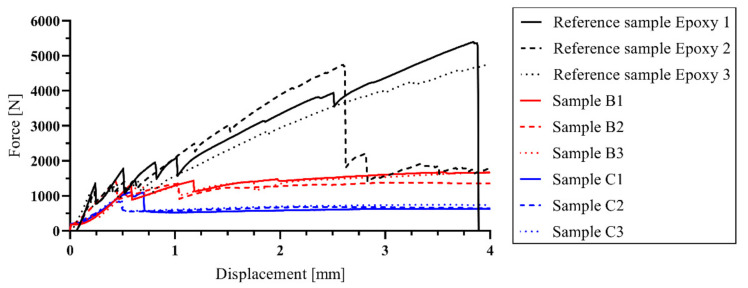
Evaluation of four-point bending tests of experimental samples from series A, B and C.

For the reasons described above, textile reinforcement saturation with CEM 52.5 R proves to be a possible variant usable for replacing the combustible synthetic matrix of textile concrete. When the reference samples and series B samples were compared, it was clear that the series B samples achieved approximately 30% efficiency of loading capacity (Figure 15). However, there could be a theoretical possibility to reach a loading capacity near to 100% by using more tensile reinforcement. It is evident from the mechanical test results (Figure 14) that the B series samples showed a similar trend of bending capacity increase, mainly in the initial phases of crack development, as has been shown in reference samples Epoxy 1. However, due to an alternative material used in this study, the results of this solution deviate from that of conventional TRC with an epoxy resin matrix [44,45,46] and a comparison of results is therefore problematic. The best-aperforming combinations (sample series A and B) will be further investigated by mechanical tests focusing on a detailed description of cement matrix behavior, for example the so-called pullout test based on simple co-interaction of one cement-saturated carbon roving with concrete. Furthermore, once sufficient performance has been determined at normal temperature, tests at elevated temperatures will be carried out.

The next steps in future work are to try using different methods of saturation to achieve full saturation of textile reinforcement. This includes a verification of the data measured from the mechanical tests on experimental samples exposed to elevated temperature according to ISO 834.

## Figures and Tables

**Table 1:** HPC component mixture of [4].

Mixture Components	Quantity
	kg∙m^−3^
CEM I 42.5 R	680
Silica sand	960
Silica flour (ground quartz)	325
Silica fume (microsilica)	175
Superplasticizer	29
Water	171
Total	2420

**Table 2:** Material data of carbon roving [31].

Material Properties	Value	Units
Sizing properties	F13	-
Number of filaments	24 000	-
Nominal linear density	1600	tex
Filament diameter	7.0	µm
Density	1.77	g·cm^−3^
**Tensile properties**		
Tensile strength	4000.0	MPa
Modulus of elasticity	240.0	GPa

**Table 3:** Variants of experimental samples.

Description of Samples	Type of Saturation
A	Experimental samples saturated with CEM 42.5 R–pressure saturation.
B	Experimental samples saturated with CEM 52.5 R–pressure saturation.
C	Experimental samples saturated with CEM 42.5 R–surface saturation by a roller.
D	Experimental samples saturated with CEM 52.5 R–surface saturation by a roller.
E	Experimental samples without any saturation.
-	Reference samples with epoxy resin matrix–surface saturation by a roller.

## Data Availability

The data presented in this study are available on request from the corresponding author.
